# Emerging therapeutics

**DOI:** 10.1186/s12967-021-02864-9

**Published:** 2021-05-05

**Authors:** Gennaro Ciliberto

**Affiliations:** IRCCS National Cancer Instituto Regina Elena, Rome, Italy

We are pleased to announce the launch of a new section in the Journal of Translational Medicine entitled “Emerging Therapeutics”. Scientific and technological advances are the necessary components in increasing our arsenal of weapons against complex diseases such as Cancer, Neurodegenerative and Cardiovascular diseases, Diabetes, Obesity as well as Infectious agents and, why not, also Aging. These days one of the most striking examples of emerging and highly successful technology is represented by the success of new genetic vaccines such as mRNA vaccines. With unprecedented short timelines, mRNA based vaccines have been able to demonstrate excellent safety and efficacy profiles in COVID-19 trials where they have received accelerated emergency approval, and are helping us overcome the SARS-CoV-2 pandemic crisis [1, 2]. Hence, emerging therapeutics have received intense research efforts and high medical interest and it is for these reasons that a dedicated space has been provided by the Journal of Translational Medicine.

In the last few decades, we have witnessed the advent of several therapeutic breakthroughs based on the development of new technologies. Particularly worth mentioning is the generation, clinical development and approval of hundreds of monoclonal antibodies directed against a variety of targets both as surface molecules or as hormones and cytokines. Aside from the so called first generation “naked” antibodies, more recent developments have been moving in several new directions such as drug conjugated antibodies, bispecific antibodies, BiTes, nanobodies, Fc modified monoclonals, etc., consequently opening up a diversified array of therapeutic applications [3–5]. A deeper understanding of our immune system and of the mechanisms underlying target recognition by antibodies, combined with genetic engineering and cellular therapies, have been at the basis of the recent developments of CAR-Ts. CAR-Ts represent powerful weapons against cancers showing unmatched efficacy as well as duration of therapeutic responses. Although the currently approved CAR-Ts find their applications at the moment exclusively in hematological cancers, further challenges are represented by applications in the treatment of solid tumors where we expect to see major research investments made in the near future [6–8]. Yet another field of intense study is represented by gene therapy with viral vectors as well as, as mentioned above, by RNA and DNA therapeutics, where the development of nanotechnologies for the delivery of nucleic acids has been making huge progress in the few last years [9–12]. Also, in the near future we expect to see a growing number of clinical applications of CRISPR/CAS9 gene editing to the therapy of both inherited and acquired diseases, an approach which shows great promise as it was recognized by the Nobel Prize in chemistry 2020 award winners Emmanuelle Charpentier and Jennifer Doudna [13, 14]. Finally our increasing understanding of the importance of microbiota in disease pathogenesis and response to therapy is opening up novel therapeutic avenues. Hence we can expect an entire new wave of microbiota-modulating approaches based either on the delivery of selected bacterial strains or, alternatively, the identification of molecules capable to modify gut microbiota composition for the benefit of patients [15–17].

In addition to what is listed above, the next wave of “emerging therapeutics” will arise not only from the development of new technologies for drug development but also from the discovery of new intervention targets and the possibility for new drug combinations. In this regard, the extensive use of ‘omics approaches, including next generation sequencing, single cell sequencing, proteomics and metabolomics combined with data interpretation by high performance computing, molecular modeling and new algorithms of artificial intelligence is expanding day by day, a universe of targets to be tackled by conventional drug discovery or, alternatively, as discussed above, with the help of new technologies [18–20].

In this highly stimulating scenario, we will be delighted for our section to be considered for publication in the Journal of Translational medicine original papers, review and perspectives that report about advances in the field.

The Journal of Translational Medicine provides high standard peer-review process and represents a platform for efficiently communicating up-to-date results and scientific discussions. The new section on “Emerging Therapeutics” will guarantee high quality and competitive publications. The Editorial Board is looking forward to receiving your contributions.
